# Consumption of avocado and associations with nutrient, food and anthropometric measures in a representative survey of Australians: a secondary analysis of the 2011–2012 National Nutrition and Physical Activity Survey

**DOI:** 10.1017/S0007114521003913

**Published:** 2022-09-14

**Authors:** Vivienne X. Guan, Elizabeth P. Neale, Yasmine C. Probst

**Affiliations:** 1 School of Medicine, Faculty of Science, Medicine and Health, University of Wollongong, Wollongong, NSW 2522, Australia; 2 Illawarra Health and Medical Research Institute, University of Wollongong, Wollongong, NSW 2522, Australia

**Keywords:** Avocado, Obesity, National Survey, Australia

## Abstract

Avocados are a rich source of nutrients including monounsaturated fats, dietary fibre, potassium and Mg, as well as phytochemicals. However, no epidemiological analysis for the associations between avocado consumption and participant anthropometric measures has been conducted in Australia. The present study aimed to perform a secondary analysis of the 2011–2012 National Nutrition and Physical Activity Survey (NNPAS) to quantify avocado consumption in the Australian population and explore the associations between avocado intakes, consumption of nutrients and food groups based on the Australian Dietary Guidelines and anthropometric measurements. Usual avocado consumption in the 2011–2012, NNPAS was determined using the multiple source method regression model. The relationship between avocado consumption and intakes of key nutrients and food groups and participant weight, BMI and waist circumference were examined using linear regression. Mean avocado intake was 2·56 (95 % CI: 2·37, 2·75) grams per day with 15·9 % of Australians considered to be ‘avocado consumers’ (*n* 21 526 456 population size; *n* 12 153 observations). Greater consumption (g) of avocados was associated with significantly higher consumption of monounsaturated fats, polyunsaturated fats, dietary fibre, vitamin E, Mg and potassium, as well as ‘whole grains’, ‘vegetables’, ‘fruit’ and ‘meat and alternatives’ food groups. Greater consumption (g) of avocados was associated with significantly lower consumption of carbohydrates and discretionary foods. When adjusted for covariates, greater consumption of avocados was significantly associated with a lower body weight (*P* = 0·034), BMI (*P* < 0·001) and waist circumference (*P* < 0·001). Avocados may be incorporated into an eating pattern and may be beneficial in weight management.

Overweight and obesity continue to grow as a global epidemic^(1,2)^. The prevalence of obesity has almost tripled since 1975^(3)^. The current prevalence of overweight and obesity among adults in Australia is 67 %^(4)^ and 52 % worldwide^(3)^. Being overweight is a risk factor for many chronic diseases (e.g. CVD) and is associated with increased mortality^(5)^. Dietary intake is a modifiable lifestyle factor for overweight and obese individuals^(6,7)^.

Avocados are a rich source of key nutrients including MUFA, dietary fibre, potassium, Mg and phytochemicals^(8,9)^. Epidemiological evidence suggests that avocado consumers have a significantly higher diet quality than non-consumers^(10)^. Avocado consumers are also reported to have significantly lower body weight and BMI than non-consumers^(10,11)^. Research has suggested that the high fat and fiber composition of avocados may contribute to weight loss by maintaining satiety and changing gut-microbiota composition^(12)^. A study has also found that isoenergetic substitution of foods with avocados in a lunch meal resulted in an increased post-ingestive satiety during a 5-h period in overweight and obese adults^(13)^. Research also suggests that including avocados in a moderate fat and cholesterol-lowering pattern of eating may contribute to additional improvements in blood lipids in overweight and obese individuals^(14)^. However, research on the health benefits of avocados in the Australian context is limited. The aim of the study was, therefore, to perform a secondary analysis of the 2011–2012 the National Nutrition and Physical Activity Survey (NNPAS) to quantify avocado consumption in the Australian population and explore the associations between avocado intakes, consumption of nutrients and food groups and anthropometric measurements of the participants.

## Methods

### Study population

The present analysis used the 2011–2012 NNPAS from the Confidentialised Unit Record Files. The 2011–2012 NNPAS, as a component of the Australian Health Survey 2011–2013, was a cross-sectional, nationally representative survey conducted by the Australian Bureau of Statistics^(15)^. The survey sampled householders in private dwellings in all states and territories (*n* 8) applying a multistaged, stratified-area, probability sampling design. The sample included 12 153 Australians aged 2 years and older (77 % response rate)^(15)^. Population weights were applied to ensure that the data were representative of the wider Australian population. Detailed information on the methods of the survey has been published elsewhere^(15)^. Ethics approval for conducting the household interview components of the survey was allowed under the Census and Statistics Act 1905^(15)^.

### Food and nutrient intake assessment

Dietary intake was assessed with two 24-h recalls conducted on separate days using the Automated Multiple-Pass Method^(16)^. The first 24-h recall was completed in person by all participants, with a second 24-h recall completed via telephone at least 8 days after the first interview in approximately 63 % of participants^(15)^.

Dietary intake data were analysed using the AUSNUT 2011–2013 food composition database, which applies a nested hierarchical structure of major, sub-major and minor food groups^(8)^. The key nutrients examined were total fat (g), saturated fat (g), monounsaturated fat (g), polyunsaturated fat (g), carbohydrate (g), protein (g), dietary fibre (g), vitamin E (mg), Mg (mg), Na (mg) and potassium (mg). The selection of nutrients was based on those found to vary between ‘avocado consumers’ and ‘non-consumers’ in a representative sample in the USA^(10)^. The Australian Dietary Guidelines database^(17)^ was applied to aggregate the dietary intake data to food group serves based on a published method^(18)^, including ‘grains’, ‘whole grains’, ‘vegetables’, ‘fruit’, ‘milk and alternatives’ and ‘meat and alternatives’. Intakes of discretionary food items, which are high in saturated fat, added salt, added sugars and alcohol (e.g. sugary drinks, sweet biscuits, cakes, confectionary, salty snack foods and processed meats), were identified using the Discretionary Food List^(19)^. One serve of a discretionary food or alcoholic beverage was defined as containing 600 kJ or 10 g alcohol, respectively^(6)^.

### Avocado intake estimation

Avocado (*Persea Americana*) is an almost pear shaped fruit with a green to black shiny skin and a delicate yellow flesh with a green outer hue^(17)^. Consumption of the avocado is generally without the seed or the skin. Avocado consumption was estimated using an avocado-specific database developed by our team^(20)^. The avocado-specific database was created based on the AUSNUT 2011–2013 food composition database^(17)^. The majority of nutrient data related to avocados were derived from a composite of eight samples of Hass avocados purchased in five Australian states^(21)^. Thus, it can be assumed that the Hass avocado is closely related to the AUSNUT 2011–2013 entry for the whole avocado^(17)^. The avocado-specific database represents avocados as a whole food item only, and avocado oil was excluded. Whole avocados and avocado-containing products were included in the present analyses. Avocado intakes were explored in grams per day. The percentage of a composite food product containing avocados was multiplied by the total weight of the product to determine the amount of avocado (in grams) consumed by each participant for each composite product.

### Outcome assessment

Demographic characteristics including the age, sex and education level were used. In the 2011–2012 NNPAS, the highest level of non-school educational attainment was self-reported by participants aged 15 years and over^(15)^. As education level was defined as the level of non-school education, data for participants who were currently attending school (i.e. children aged <18 years) were excluded from all regression analyses. Self-reported physical activity information was collected for participants aged 18 years and over. This information was categorised into levels of physical activity, outlined in further detail elsewhere^(15)^. Measurement of height (cm), body weight (kg) and waist circumference (cm) was recorded to one decimal place, using a stadiometer, digital scales and a metal tape measure, respectively^(15)^. BMI was calculated as weight (kg) divided by height (m) squared. Waist circumference was taken at the top of the umbilicus^(15)^.

### Statistical analysis

All statistical analyses were conducted using Stata/IC Version 15 (version 15, StataCorp, 2017). To account for the survey design, sampling process and provide unbiased estimates, replicate weightings were applied using jack-knife resampling in the present analysis^(22)^. A set of replicate weights and personal weights was supplied with the Confidentialised Unit Record Files. Commands were conducted in Stata using the complex design model, with details and the Stata codes on how to use replicate weight in NNPAS published elsewhere^(22)^.

Usual intakes of key nutrients, dietary energy and food groups were calculated from the two 24-h recalls using the Multiple Source Method^(23)^ regression model. Mean and 95 % CI and median and 25th and 75th percentiles for avocado consumption (as grams per day) were determined for all participants. Participants were categorised into sub-samples as either ‘avocado consumers’ or ‘non-consumers’, and the proportion of ‘avocado consumers’ and ‘non-consumers’ was determined. To examine avocado intakes in different population groups, the mean avocado intakes in the NNPAS sample and for ‘avocado consumers’ (as grams per day), as well as avocado consumption stratified by age group and sex, were examined. Avocado consumption was compared across age and sex categories using linear regression with pairwise comparisons of marginal linear predictions with Bonferroni adjustments. Food groups and nutrients intake were also compared between consumers and non-consumers using linear regression with pairwise comparisons of marginal linear predictions. The relationship between avocado intakes and the reported consumption of key nutrients and food groups was explored using survey-adjusted linear regression, which were adjusted for confounding using age, sex and usual energy intake (kilojoules per day). Linear regression was performed to explore the association between avocado consumption and anthropometric measures (weight, BMI and waist circumference), which were adjusted for confounding using age, sex usual energy intake (kilojoules per day) and physical activity. Directed acyclic graphs were created to guide the selection of covariates for the analysis^(24)^ (online Supplementary Fig. 1). Linearity, homoscedasticity and normality of residuals were checked for all variables for all regression analyses. A two-tailed *P*-value < 0·05 was considered significant.

## Results

### Avocado intake

Among Australians overall, the mean reported avocado intake was 2·56 (95 % CI: 2·37, 2·75) grams per day. Median avocado consumption was 0·00 (25th and 75th percentiles: 0·00–0·00) grams per day. From the NNPAS results, 15·9 % of Australians were found to be ‘avocado consumers’ and 84·1 % were ‘non-consumers’. When the analysis was limited to ‘avocado consumers’ only, the mean avocado intake was 16·09 (95 % CI: 15·41, 16·78) grams per day, with a median intake of 11·37 (25th and 75th percentiles: 6·84–21·19) grams per day.

For all participants, the highest avocado intakes were in adults aged 18–64 years (mean: 2·37 g, 95 % CI: 2·02, 2·72 for males; mean: 3·70 g, 95 % CI: 3·37, 4·04 for females) (Table 1). For ‘avocado consumers’, there were no significant differences in avocado intake among males across the age groups. For female ‘avocado consumers’, those aged 18–64 years consumed significantly higher amounts of avocados than older adults (*P* = 0·044), with no significant differences seen between children. The highest avocado intake among ‘avocado consumers’ was observed in children 2–8 years (mean: 19·07 g (95 % CI: 11·02, 27·12) for males, mean: 19·24 g (95 % CI: 12·96, 25·52) for females). Among ‘avocado consumers’, there was no significant difference in avocado intakes between education levels (Table 2).

**Table 1.** Mean (95 % confidence interval) and median (25th and 75th percentiles) avocado consumption by sex and age groups, for all Australians, and ‘avocado consumers’ only, 2011–2012 National Nutrition and Physical Activity Survey (NNPAS)*,†,‡ (Mean values and 95 % confidence intervals; median values and percentiles)

	All participants						‘Avocado consumers’					
	Population size	Numbers of observations	Avocado intake (g)		Median (25th and 75th percentiles) avocado intake (g)		Population size	Numbers of observations	Avocado intake (g)		Median (25th and 75th percentiles) avocado intake (g)	
Sex and age group			Mean	95 % CI	Median	25th and 75th percentiles			Mean	95 % CI	Median	25th and 75th percentiles
Males (years)												
Children (2–8)	1 002 059	625	0·95	0·33, 1·56	0	0, 0	49 682	25	19·07	11·02, 27·12	12·68	6·05, 31·26
Children (9–13)	770 917	392	0·63	0·14, 1·12	0	0, 0	38 725	15	12·57	6·91, 18·22	12·64	6·05, 18·29
Children (14–17)	528 535	356	0·77	0·26, 1·28	0	0, 0	24 801	20	16·35	10·22, 22·47	10·90	9·08, 21·37
Adults (18–64)	7 031 494	3419	2·37	2·02, 2·72	0	0, 0	1 050 199	513	15·84	14·29, 17·40	10·90	7·26, 21·19
Older adults (65 and over)	1 374 767	910	2·16	1·53, 2·80	0	0, 0	218 567	148	13·61	11·39, 15·83	7·92	6·05, 18·03
Females (years)												
Children (2–8)	931 442	628	0·85	0·35, 1·36	0	0, 0	41 374	30	19·24	12·96, 25·52	18·18	9·80, 27·40
Children (9–13)	755 089	395	0·77	0·32, 1·23	0	0, 0	48 766	32	11·99	7·05, 16·94	6·84	5·16, 21·19
Children (14–17)	496 206	322	1·65	0·88, 2·42	0	0, 0	48 321	34	16·94	12·86, 21·01	15·91	6·84, 21·19
Adults (18–64)	7 090 526	3913	3·70	3·37, 4·04	0	0, 0	1 533 277	816	17·13	16·07, 18·19†	12·88	7·58, 21·19
Older adults (65 and over)	1 545 421	1193	3·38	2·78, 3·99	0	0, 0	372 461	265	14·03 (	12·28, 15·78†	10·78	6·84, 21·19

* Complex design using a jack-knife procedure with the replicate weights.

† Australians: population size: 21 526 456, number of observations: 12 153; ‘avocado consumers’: population size: 3 426 173, number of observations: 1898.

‡ Indicate significant differences (*P* < 0·05) between groups after Bonferroni adjustment.

**Table 2.** Mean (95 % confidence interval) and median (25th and 75th percentiles) avocado consumption by education level, for ‘avocado consumers’ only, 2011–2012 National Nutrition and Physical Activity Survey (NNPAS)*,† (Mean values and 95 % confidence intervals; median values and percentiles)

Education level	Population size	Numbers of observations	Avocado intake (g)		Avocado intake (g)	
			Mean	95 % CI	Median	25th and 75th percentiles
Postgraduate Degree, Graduate Diploma/Graduate Certificate	326 768	197	16·27	13·43, 19·10	11·32	6·84, 21·19
Bachelor Degree, Advanced Diploma/Diploma	1 046 209	581	16·89	15·46, 18·32	11·91	7·58, 21·19
Certificate (Certificate III/IV, Certificate I/II, Certificate not further defined)	634 436	345	16·00	14·36, 17·64	10·90	6·84, 21·19
No non-school qualification	1 195 483	642	15·50	14·32, 16·69	10·90	6·84, 21·19

* Complex design using a jack-knife procedure with the replicate weights.

† Australians: population size: 21 526 456, number of observations: 12 153; ‘avocado consumers’: population size: 3 426 173, number of observations: 1898.

### Associations between avocado intake and dietary intakes

Compared with non-consumers, the intakes of avocado consumers were significantly higher in monounsaturated fats, polyunsaturated fats, dietary fibre, vitamin E, Mg and potassium (all *P* < 0·001) (Table 3). Avocado consumers had significantly higher intakes of ‘whole grains’ (*P* < 0·001), ‘vegetables’ (*P* < 0·001), ‘fruit’ (*P* = 0·002) and ‘meat and alternatives’ (*P* = 0·004) and lower intake of ‘grains’ (*P* < 0·001), ‘milk and alternatives’ (*P* = 0·012) and ‘discretionary foods’ (*P* < 0·001).

**Table 3.** Average (95 % confidence interval) daily nutrient intakes and serving intakes of the Australian dietary guidelines food groups for avocado consumers and non-consumers*,† (Average and 95 % confidence intervals)

	Consumers		Non-consumers		
	*n* 3 426 173 (obs:1898)		*n* 18 100 283 (obs:10 255)		
	Average	95 % CI	Average	95 % CI	*P* value
Nutrients					
Total energy (MJ)	6·82	6·71, 6·94	6·89	6·83, 6·95	0·315
Total fat (g)	59·94	58·82, 61·06	58·30	57·69, 58·91	0·009
Saturated fat (g)	21·75	21·26, 22·23	22·72	22·46, 22·98	0·001
Monounsaturated fat (g)	23·43	22·96, 23·90	22·03	21·77, 22·30	< 0·001
Polyunsaturated fat (g)	9·55	9·33, 9·77	8·63	8·54, 8·73	< 0·001
Carbohydrate (g)	171·97	168·15, 175·79	186·95	185·25, 188·66	< 0·001
Protein (g)	72·44	71·02, 73·85	71·50	70·76, 72·24	0·259
Dietary fibre (g)	19·35	18·86, 19·84	18·01	17·80, 18·21	< 0·001
Vitamin E (mg)	9·11	8·90, 9·33	7·95	7·84, 8·05	< 0·001
Ca (mg)	662·44	644·02, 680·85	654·96	647·21, 662·71	0·442
Mg (mg)	281·67	275·89, 287·46	256·51	253·66, 259·37	< 0·001
Na (mg)	1874·33	1829·77, 1918·90	1957·26	1939·04, 1975·48	0·001
Potassium (mg)	2444·15	2397·16, 2491·14	2270·65	2247·58, 2293·71	< 0·001
Food groups					
Grains (serves)	4·14	4·05, 4·23	4·50	4·45, 4·55	< 0·001
Whole grains (serves)	1·49	1·44, 1·53	1·28	1·26, 1·30	< 0·001
Vegetables (serves)	2·84	2·75, 2·93	2·58	2·54, 2·61	< 0·001
Fruit (serves)	1·46	1·41, 1·52	1·36	1·34, 1·38	0·002
Milk and alternatives (serves)	1·37	1·32, 1·42	1·44	1·41, 1·46	0·012
Meat and alternatives (serves)	2·13	2·07, 2·19	2·03	2·00, 2·05	0·004
Discretionary foods (serves)	3·66	3·56, 3·77	3·99	3·56, 3·77	< 0·001

* Complex design using a jack-knife procedure with the replicate weights.

† Australians: population size: 21 526 456, number of observations: 12 153.

**Table 4.** Linear regression for avocado consumption (g), nutrients and food groups using the 2011–2012 NNPAS*,†,‡ (Coefficient and 95 % confidence intervals)

	Coefficient	95 % CI	*t*	*P* value
Nutrients				
Total fats (g)	0·117	0·088, 0·146	8·11	< 0·001
Saturated fat (g)	–0·017	–0·035, 0·001	–1·86	0·068
Monounsaturated fats (g)	0·084	0·071, 0·098	12·57	< 0·001
Polyunsaturated fats (g)	0·038	0·029, 0·048	8·18	< 0·001
Carbohydrates (g)	–0·442	–0·542, −0·342	–8·85	< 0·001
Protein (g)	0·034	–0·010, 0·079	1·55	0·128
Dietary fibre (g)	0·060	0·040, 0·080	6·00	< 0·001
Vitamin E (mg)	0·042	0·0341, 0·0506	10·32	< 0·001
Ca (mg)	0·574	–0·183, 1·330	1·52	0·134
Mg (mg)	0·876	0·674, 1·078	8·67	< 0·001
Na (mg)	–1·659	–3·326, 0·007	–1·99	0·051
Potassium (mg)	6·043	4·612, 7·474	8·45	< 0·001
Food groups				
Grains (serves)	–0·007	–0·010, −0·003	–3·59	0·001
Whole grains (serves)	0·009	0·006, 0·011	6·04	< 0·001
Vegetables (serves)	0·013	0·009, 0·017	6·85	< 0·001
Fruit (serves)	0·004	0·001, 0·006	2·79	0·007
Milk and alternatives (serves)	–0·001	–0·004, 0·001	–1·36	0·180
Meat and alternatives (serves)	0·003	0·001, 0·005	2·61	0·012
Discretionary foods (serves)	–0·017	–0·023, −0·012	–6·17	< 0·001

* Complex design using a jack-knife procedure with the replicate weights.

† Australians: population size: 21,526,456, number of observations: 12 153; ‘avocado consumers’: population size: 3 426 173, number of observations: 1898.

‡ Adjusted for age, sex and usual energy intake (kilojoules per day).

After adjusting for covariates, greater consumption of grams of avocados was associated with significantly higher consumption of monounsaturated fats (*P* < 0·001), polyunsaturated fats (*P* < 0·001), dietary fibre (*P* < 0·001), vitamin E (*P* < 0·001), Mg (*P* < 0·001) and potassium (*P* < 0·001), as well as ‘whole grains’ (*P* < 0·001), ‘vegetables’ (*P* < 0·001), ‘fruit’ (*P* = 0·007) and ‘meat and alternatives’ (*P* = 0·012) food groups. Non-significant associations were found between avocado consumption and saturated fats (*P* = 0·068), protein (*P* = 0·128), Ca (*P* = 0·134), Na (*P* = 0·051) and ‘milk and alternatives’ (*P* = 0·180). Greater consumption of avocados was associated significantly to lower consumption of carbohydrates and discretionary foods (all *P* < 0·001).

### Associations between avocado consumption and anthropometric measures

**Fig. 1.**
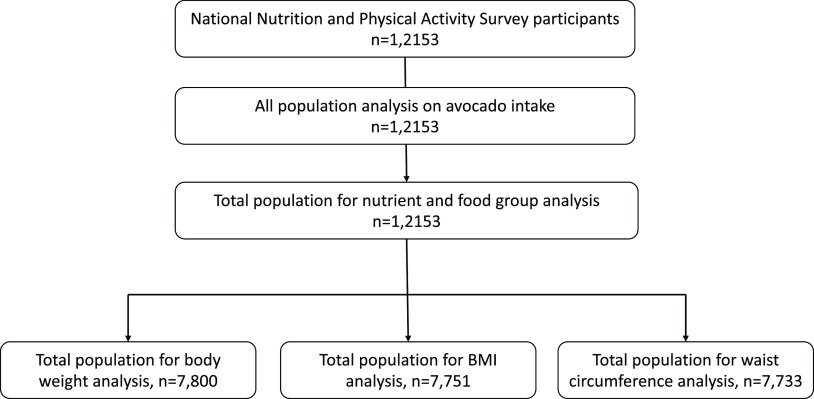
Participant flowchart for secondary analysis of the Australian Health Survey, National Nutrition and Physical Activity Survey component

The associations between avocado intake and anthropometric measures were explored in participants aged 18 years and older (Fig. 1). When adjusted for covariates, greater consumption of avocados was significantly associated with a lower body weight (kg) (coefficient: –0·05, 95 % CI: –0·11, –0·01, *t*: –2·17, *P* = 0·034), BMI (kg/m^2^) (coefficient: –0·03, 95 % CI: –0·04, –0·01, *t*: –3·64, *P* < 0·001) and waist circumference (cm) (coefficient: –0·07, 95 % CI: –0·10, –0·03, *t*: –3·83, *P* < 0·001).

## Discussion

To our knowledge, this study is the first to examine avocado consumption among Australians using a representative national survey. This is also the first study to investigate the associations between avocado consumption and anthropometric outcomes. A novel avocado-specific database was applied to assess avocado consumption from all food sources including those foods in mixed dishes. This research offers insights into avocado consumption in Australia, as well as associations between the intakes of avocados, key nutrients and food groups based on the Australian Dietary Guidelines.

The present study highlighted the importance of developing an avocado-specific database to accurately estimate avocado intake. The proportion of avocado consumers (defined as consuming the whole avocado only) in the USA population (≥ 19 years of age) was suggested to be approximately 2 % (*n* 347), which was identified from several cycles of NHANES (2001–2008)^(10)^. The discrepancy in the finding between the present analysis and the USA cohort may be due to the food composition data used to estimate the avocado intakes. In the present study, avocados were consumed not only as whole fruit but also from composite (multi-ingredient) foods, such as guacamole, sushi, hamburgers and Mexican style dishes. The avocado intake, therefore, appears to be underestimated by limiting the measurement of avocado consumption to the whole avocado alone. Thus, to facilitate an accurate estimation of avocado intake, a systematic approach is required to include specific information for avocado and avocado-containing foods, including composite foods.

The findings show that avocado consumption was associated with favourable intakes of key nutrients and food groups based on the Australian Dietary Guidelines. These favourable nutrient intakes are consistent with previous epidemiological and clinical data^(10,25–27)^. Although avocado consumers had higher intakes of fruit and vegetables as suggested by the present analysis and the USA cohort analysis, Fulgoni et al reported that there were no significant differences between avocado consumers and non-consumers for intakes of total grains, whole grains, dairy, meat and beans and discretionary fats in the USA cohort^(10)^. The differences in the food supply and definitions of food groups between Australia and the USA may contribute to these differences in food group intakes. Further, the literature has suggested that education level may be positively associated with diet quality^(28)^. While diet quality was not specifically addressed in the present study, the lack of an association between avocado intake and education level is of interest as a previous analysis of the NNPAS has indicated that Australians with higher education levels consume higher intakes of nuts, another core food^(29)^. It is, therefore, worthwhile that the relationship between avocado intakes and education level is further investigated. Avocados are a good source of MUFA, dietary fibre, potassium and Mg^(8,9)^. Given energy intake was not different between consumers and non-consumers identified by literature^(10)^ and the present analysis, avocado consumption may be an indicator of improved diet quality. This may in part be due to the contribution of the avocados to intakes of key nutrients. It is also possible that the consumption of avocados may facilitate the consumption of other core foods, as our team has previously found for another core food, nuts^(30)^.

As avocados are suggested to be a medium energy density food item^(9)^, it is pertinent to examine the association between avocado consumption and anthropometric outcomes. The present findings suggest that avocado consumption was inversely associated with body weight, BMI and waist circumference, aligning with previous epidemiological and clinical studies^(10–12)^. The benefits of avocado consumption on weight management may be attributed to various components including dietary fiber by maintaining satiety and changing gut-microbiota composition. The literature suggests that the volume of a meal plays a role in influencing satiety^(31)^. Adding whole avocado to a meal tends to increase the meal volume similar to other fruit and vegetables^(13)^. Further, isoenergetic substitution of foods with avocados in a meal has resulted in an increased post-ingestive satiety and reduced motivation to eat in overweight and obese adults^(13,27)^. Clinical data also show that avocado consumption can increase the abundance of gut bacteria involved in plant polysaccharide fermentation in overweight and obese adults^(12,26)^. The changes in the gut bacterial composition may lead to a change in energy utilisation in the large intestine^(12)^. Avocados contains approximately 13 % fat, of which 74 % are represented as MUFA^(17)^. The fat profile of avocados may play a role in body composition among avocado consumers. The literature suggests that the intake of MUFA may contribute to stimulating the expression of leptin mRNA, which assists in regulating energetic homoeostasis^(32)^. Diets rich in MUFA appear to favour satiety and reduce food intake^(33)^. Intake of MUFA may also increase thermogenesis promoting greater energy expenditure and lower fat storage^(34)^. Therefore, it seems that the fibers and MUFA present in avocados may regulate satiety signalling and thermogenesis to favour of body weight control. In addition, avocado consumption as associated with favourable diet quality should also be considered^(10)^. As such, the inverse relationship between avocado consumption and anthropometric measures may be contributed by avocado consumers eating a healthier diet overall.

While assessing avocado consumption derived from a representative national survey has many strengths, it is not without limitations. First, the present study was a secondary analysis of a cross-section survey. Therefore, the causal evidence between avocado consumption, intakes of nutrients and food groups and anthropometric outcomes cannot be established. Second, as the proportion of avocado consumers in the analysis was small, the power of analyses may be limited. While dietary intake was collected using the Automated Multiple-Pass Method, self-report dietary intake data are prone to recall bias. The data may be biased due to over-reporting or under-reporting foods to conform to societal norms. It should be noted that this study did not exclude respondents who were considered to be misreporting their intakes. To ensure that avocado intakes could be compared across the population, avocado consumption was presented in grams rather than as a percentage of energy. It should be noted that comparison of consumptions across age and sex groups may be affected by total energy intakes. Finally, although directed acyclic graphs were used to guide the decision of covariates for analyses, it is possible that other confounding variables were present that may have affected the results. Therefore, the findings of the present analysis should be interpreted accordingly.

### Conclusion

This study is the first to explore avocado consumption in a representative Australian sample which accounted for avocado from all sources. The use of a national representative estimate makes it possible to generalise findings to the population at large. The findings suggest that there were significant associations between avocado consumption and a higher intakes of key nutrients and food groups based on the Australian Dietary Guidelines intakes and lower body weight, BMI and waist circumference. These results suggest that the incorporation of avocado in the diet may support favourable diet quality and be associated with a lower body weight, BMI and waist circumference.

## Supplementary material

For supplementary material accompanying this paper visit https://doi.org/10.1017/S0007114521003913. click here to view supplementary material
