# Supplementary figures and images for: Consumption of avocado and associations with nutrient, food and anthropometric measures in a representative survey of Australians: a secondary analysis of the 2011–2012 National Nutrition and Physical Activity Survey
Source: Br J Nutr. 2021 Sep 29;128(5):932–9. doi: 10.1017/S0007114521003913 (PMC9361124; doi:10.1017/S0007114521003913)

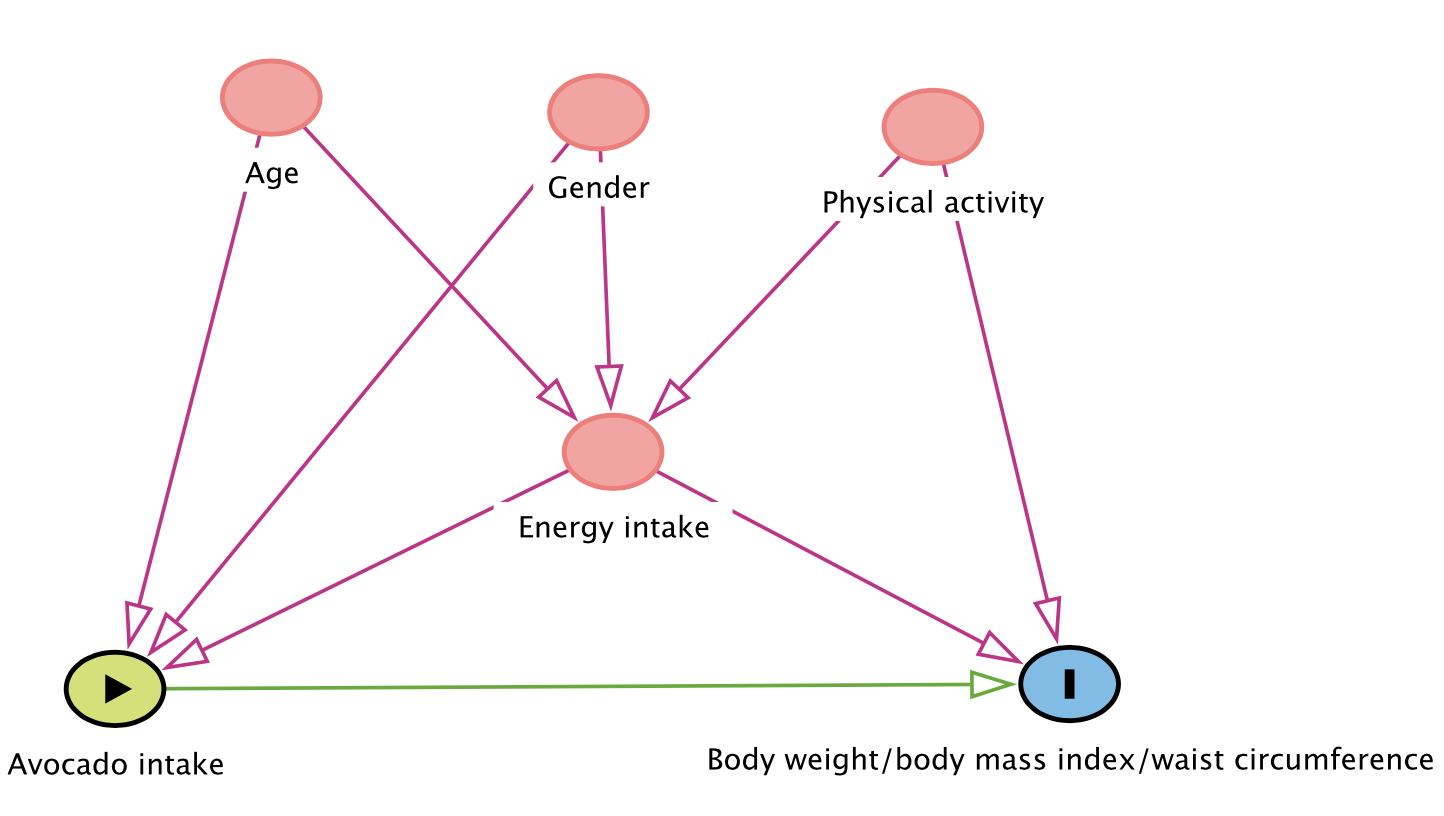


Supplementary Figure 1

Supplement: Supplementary file 1 [file S0007114521003913sup001.docx]
